# Effect of follow-up period on minimal-significant dose in the atomic-bomb survivor studies

**DOI:** 10.1007/s00411-017-0720-7

**Published:** 2017-11-21

**Authors:** John Cologne, Dale L. Preston, Eric J. Grant, Harry M. Cullings, Kotaro Ozasa

**Affiliations:** 1 Radiation Effects Research Foundation, 5-2 Hijiyama Park, Minami-ku, Hiroshima, 732-0815 Japan; 2 Hirosoft International, 1335 H St., Eureka, CA 95501 USA

**Keywords:** Atomic-bomb survivors, Life Span Study, Low-dose research, Risk assessment

## Abstract

It was recently suggested that earlier reports on solid-cancer mortality and incidence in the Life Span Study of atomic-bomb survivors contain still-useful information about low-dose risk that should not be ignored, because longer follow-up may lead to attenuated estimates of low-dose risk due to longer time since exposure. Here it is demonstrated, through the use of all follow-up data and risk models stratified on period of follow-up (as opposed to sub-setting the data by follow-up period), that the appearance of risk attenuation over time may be the result of less-precise risk estimation—in particular, imprecise estimation of effect-modification parameters—in the earlier periods. Longer follow-up, in addition to allowing more-precise estimation of risk due to larger numbers of radiation-related cases, provides more-precise adjustment for background mortality or incidence and more-accurate assessment of risk modification by age at exposure and attained age. It is concluded that the latest follow-up data are most appropriate for inferring low-dose risk. Furthermore, if researchers are interested in effects of time since exposure, the most-recent follow-up data should be considered rather than the results of earlier reports.

**Electronic supplementary material:**

The online version of this article (10.1007/s00411-017-0720-7) contains supplementary material, which is available to authorized users.

## Introduction

Walsh and Schneider (2016), in a re-analysis of the A-bomb survivor Life Span Study (LSS) solid-cancer mortality (Ozasa et al. 2012) and incidence (Preston et al. 2007) data, concluded that the minimal-significant dose range (i.e. the lowest dose level at which one can obtain a statistically significant estimate of the slope parameter for a linear dose response on the interval from zero to that dose) has been increasing with progressive follow-up—in other words, that longer follow-up has led to attenuation or weaker evidence of low-dose effects. Walsh and Schneider concluded that the reason for this could be decreasing risk with time since exposure, which means that longer follow-up includes longer periods with lower risk. They stated, “Some evidence was provided that results on low-dose radiation risks from earlier follow-up periods should not be ignored once the results from the new follow-ups are published”. Although a decline in risk with time since exposure would be a natural consequence of the decrease in risk with age that we observe as the LSS—a fixed cohort—ages (because all cohort members were exposed at the same time, attained age and time since exposure are collinear), the suggestion that earlier reports may have more reliable low-dose information seems contrary to intuition. Longer follow-up should lead to increased power for low-dose risk estimation, increased accuracy of estimated effects on the risk of age and time, and improved precision of background-rate estimation.

Walsh and Schneider reached their conclusion by comparing risks estimated with different subsets of the data selected according to period of follow-up, corresponding closely to what was actually done in the series of LSS reports. Such sub-setting produces data sets of different size in terms of numbers of cases and person years; the results with different follow-up periods are therefore not strictly comparable because there will be variation among periods in estimates of the overall background mortality or incidence, and the slope of the dose response is dependent on the intercept (the overall or average rate; Cologne and Preston 2001). There will also be variation among periods in the precision and accuracy of estimates of effect-modification parameters. To overcome these limitations, all of the latest follow-up data were used here for estimation of background rates and effect-modification parameters, and the low-dose-range slope of the excess relative risk (ERR) was analysed according to follow-up period by stratifying the dose response on period. The minimal-significant dose level (*D*_min_) was also calculated using an objective definition of minimal significance that is not susceptible to declaring a statistically significant dose range from isolated low-dose results (i.e., large risk estimates in a few scattered low-dose categories) that may arise due to chance with small numbers of person-years of observation and relatively large variability in the number of radiation-related excess cancer cases.

## Materials and methods

The Life Span Study (LSS) of atomic-bomb survivors is well documented (Ozasa et al. 2012; Grant et al. 2017), so prior knowledge of the study and methods is assumed. Results of analyses of solid cancer incidence and mortality have appeared in a series of reports, generally over about 10-year intervals for incidence and 5-year intervals for mortality. As pointed out by Walsh and Schneider (2016), results may vary somewhat from report to report, which is a natural consequence of repeated analyses of accumulating data from ongoing follow-up. An obvious limitation of comparing results from different reports is that some aspects of the fitted models, such as background rates and effect-modification parameters, can be estimated more accurately and more precisely with greater follow-up. Thus, an assessment of the impact of follow-up period (or time since exposure) on low-dose risk should be based on all of the available data through the latest follow-up, and this is what was done here.

The standard approach to LSS analyses was used—i.e., the rate (mortality or incidence) was modeled using a product of two model terms, one for the background rate and one for the ERR. Our model was:

$$\lambda (c,s,a,b,g,d,e)={\lambda _0}(c,s,a,b,g) \times [1+{\text{ERR}}(d,a,e,s)],$$

where *c* is city, *s* is sex (coded as − 1 for males and + 1 for females), *a* is attained age in years, *b* is birth year, *g* is ground distance from the hypocenter (which was not included in previous LSS analyses; see below), *d* is radiation dose, and *e* is age in years at the time of exposure (please refer to the LSS reports for further details: Ozasa et al. 2012; Grant et al. 2017).

To investigate the impact of follow-up period on low-dose risk and to calculate the minimal-significant dose level *D*_min_, a disjoint two-segment linear model was used to estimate the ERR and its 95% likelihood-based confidence interval for a linear dose response up to each candidate minimal-significant dose level and this segmented dose response was further stratified by follow-up period (before or after the end of follow-up for each major LSS report). Therefore, four dose–response ERR parameters were estimated, one for each combination of two dichotomous factors: below or above the dose-range cutoff and prior to or after the end of the follow-up period under consideration. With effect modification handled in the usual way, the resulting ERR dose–response model is defined as:

$${\text{ERR}}=\left[ {\left( {{\beta _{L1}}{d_L}+{\beta _{U1}}{d_U}} \right){\delta _P}+\left( {{\beta _{L2}}{d_L}+{\beta _{U2}}{d_U}} \right)(1 - {\delta _P})} \right] \times \left[ {{e^{\tau (e - 30)+\upsilon \ln (a/70)}}\left( {1+\sigma s} \right)} \right],$$

where *d*_L_ (*d*_U_) is the dose if $d<{d_j}\,\,\left( {d \geqslant {d_j}} \right)$ and zero otherwise, for cutpoints (candidate minimal-significant dose levels) *d*_*j*_ that correspond to the LSS person-year tabulation {0.005, 0.02, 0.04, 0.06, 0.08, 0.1, 0.125, 0.15, 0.175, 0.2, 0.25, 0.3, 0.5, 0.75, 1.0, 1.25, 1.5, 1.75, 2.0, 3.0 Gy}, and ${\delta _P}={I_{T \in P}}$ is an indicator of whether the calendar time *T* of a Poisson observation (person-year cell) in the person-year data table is in the follow-up period *P* of interest (taking value 1 if yes, 0 if no). Period *P* was defined to be 1950 up to 1985, 1990, 1995, or 2003 for solid cancer mortality and 1958 up to 1987, 1998, or 2009 for solid cancer incidence. Note that there is no splitting of the dose response on period with the most-recent mortality follow-up period or the most-recent incidence follow-up period. In this way, the background model is estimated using the same data for each follow-up period analysis because there is no sub-setting of the data by period, but the ERR up to cutpoint *d*_*j*_ (i.e., over the dose range [0, *d*_*j*_), which means 0 ≤ *d* < *d*_*j*_) is estimated separately for the period *P* if *P* is prior to 2003 (for mortality) or 2009 (for incidence).

Background solid cancer mortality was handled with stratification on city, sex, age, and birth year (using age at the time of the bombing as a surrogate for birth year) as in Ozasa et al. (2012). We additionally stratified on two categories of ground distance from the hypocenter (proximal—within 3 km, and distal—between 3 and 10 km) at the time of the bombing (Grant et al. 2015), because of the potential for confounding given that there is a difference between the two cities in the relationship between urban/rural status (which may be correlated with unmeasured morbidity and mortality risk factors) and proximity to the hypocenter (which is correlated with radiation dose). Background solid cancer incidence was modeled parametrically as in Grant et al. (2017), with the additional inclusion of city and an interaction between city and proximal/distal ground distance. For simplicity, and to allow direct comparison with previous results, separate ERRs were not estimated for each sex, and curvature of the dose response was not considered. The Epicure AMFIT software (Risk Sciences International, Metcalfe, Canada) was used for all model fitting.

There is no clear or generally accepted definition of minimal-significant dose level *D*_min_. Ideally, one would like to declare a cutpoint *d*_*j*_ to be the *D*_min_ if all linear ERR estimates for dose ranges from 0 up to any *d* ≥ *d*_*j*_ have lower confidence bounds that exclude zero and all linear ERR estimates for dose ranges from 0 up to any *d* < *d*_*j*_ have lower confidence bounds that include zero. However, such unique cutpoints typically do not exist because of uncertainties in estimated ERR at very low doses, where there are small numbers of radiation-associated cases of cancer. Therefore, *D*_min_ is defined here to be the lowest value of *d*_*j*_ for which all linear ERR estimates for dose ranges wider than [0,*d*_*j*_) have lower confidence bounds that exclude zero. With this definition, an occasional dose range with a significant value of ERR that occurs in the midst of several ranges with non-significant values of ERR would not be considered to be the *D*_min_. The significance of such isolated instances is dependent partly on the choice of cutpoints, which is arbitrary, and may be due to chance given small numbers of radiation-related cases.

## Results

**Fig. 1:**
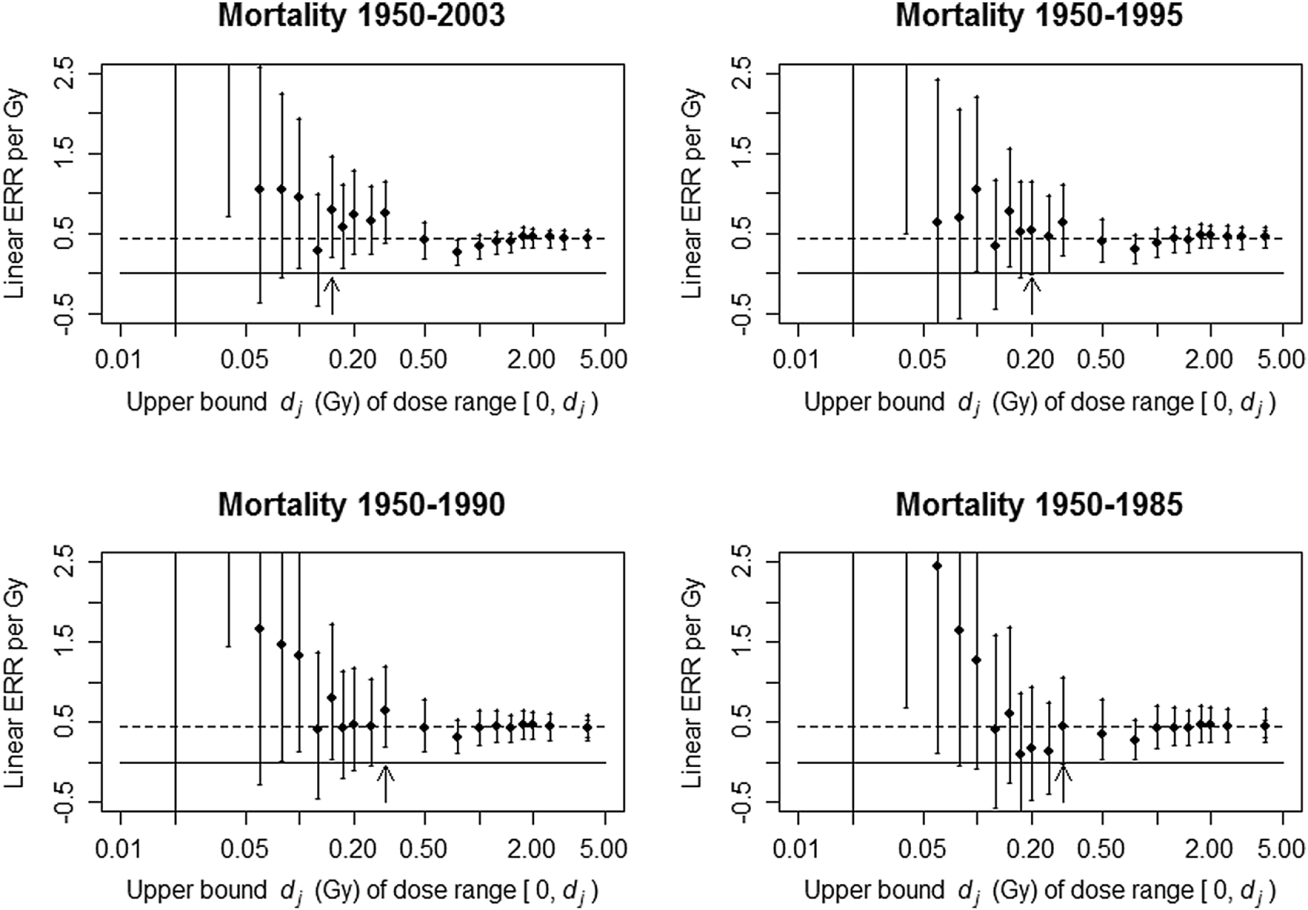
Solid cancer mortality ERR estimates and 95% likelihood-based confidence intervals for dose ranges from zero up to selected cutpoints. There were only two deaths from solid cancer above 3 Gy prior to 1990, so the segmented ERR model did not converge with *d*_*j*_ = 3.0 in the 1950–1990 and 1950–1985 periods. The point for dose range up to 4 Gy represents the ERR for the entire dose range with no segmentation in the dose response. Dashed line is the ERR estimated over the full dose range using the entire follow-up [0.438; 95% CI (0.328, 0.550)]. Solid line is at zero. Arrows demark the value of *D*_min_ in each period. The notation [0, *d*_*j*_) on the *X*-axis represents 0 ≤ *d* < *d*_*j*_

With solid cancer mortality (Fig. 1), estimates of the minimal-significant dose level became lower with longer follow-up time: *D*_min_ was 0.3 Gy for 1950–1985 and 1950–1990, 0.2 Gy for 1950–1995, and 0.15 Gy for 1950–2003, and the lower bound of the confidence interval for the dose range up to 0.3 Gy in the 1950–1985 period was close to zero (0.0001). Estimated values of linear ERR per Gy over dose ranges below about 0.1 Gy tended to be higher—and occasionally have higher values of the lower confidence bound—in the earlier follow-up periods, but these estimates did not consistently exclude the value zero from their confidence intervals. ERR estimates in dose ranges with *d*_*j*_ around 0.15 Gy (the *D*_min_ estimated with the latest follow-up) tended to be higher with longer follow-up. ERR estimates in dose ranges with *d*_*j*_ above 0.15 Gy did not show any trend with follow-up period.

**Fig. 2:**
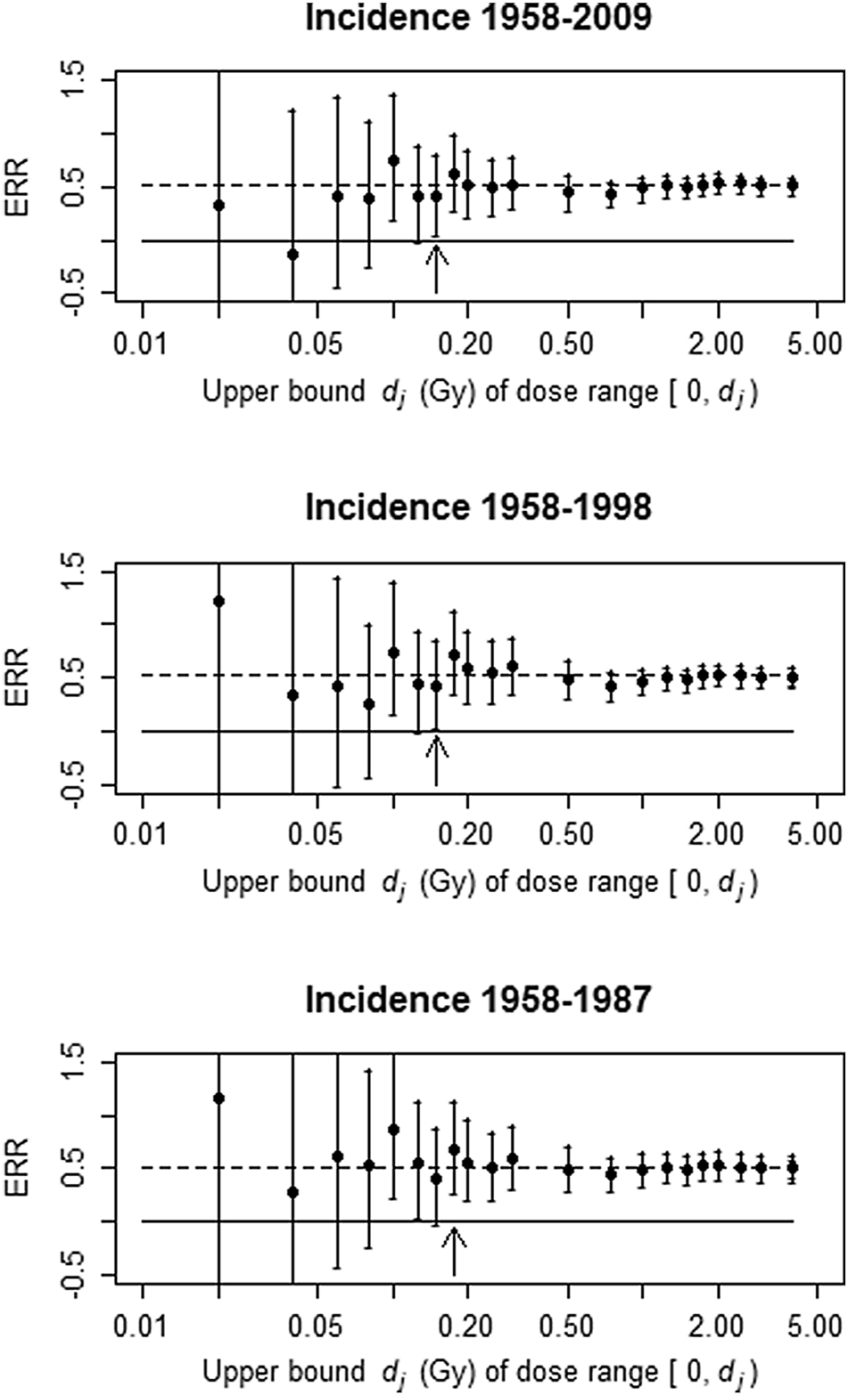
Solid cancer incidence ERR estimates and 95% likelihood-based confidence intervals for dose ranges from zero up to selected cutpoints. The point for dose range up to 4 Gy represents the ERR for the entire dose range with no segmentation in the dose response. Dashed line is the ERR estimated over the full dose range using the entire follow-up [0.516; 95% CI (0.430, 0.605)]. Solid line is at zero. Arrows demark the value of *D*_min_ in each period. The notation [0, *d*_*j*_) on the *X*-axis represents 0 ≤ *d* < *d*_*j*_

With solid cancer incidence (Fig. 2), results were qualitatively similar to those with mortality. The value of *D*_min_ was 0.175 Gy in the earliest follow-up period (1958–1987) and 0.15 Gy in the most recent two follow-up periods (1958–2009 and 1958–1998). Over the dose ranges below about 0.1 Gy, ERR estimates and their lower confidence bounds tended to be higher with shorter follow-up, but did not consistently exclude zero. Otherwise, ERR values estimated over the dose ranges did not show much variation with length of follow-up.

The background–and effect-modification–parameter estimates (based on the full data) varied little across all analyses in Figs. 1 and 2 with changing dose cutpoint and changing period segments in the dose response (not shown). To mimic the effect of data sub-setting on estimation of effect-modification parameters, the effect-modification parameters were stratified by follow-up period in the same way that the disjoint segmented ERR model was stratified (see Online Resource). This is analogous to estimating the effect-modification parameters separately for each follow-up period, except that the full data are used for background-rate estimation. The estimated full-dose-range mortality ERR tended to be higher in earlier follow-up periods when the effect-modification parameters were stratified on follow-up period: 0.544 for 1950–1985, 0.492 for 1950–1990, and 0.474 for 1950–1995. With estimation of effect modification using the full data (i.e. no stratification of effect-modification parameters on follow-up period), there was no consistent trend in the ERR by period: 0.448 for 1950–1985, 0.434 for 1950–1990, 0.454 for 1950–1995, and 0.438 for 1950–2003. With incidence, the estimated full-dose-range ERR was higher only in the earliest follow-up period when the effect-modification parameters were stratified on follow-up period—0.540 for 1958–1987 and 0.508 for 1958–1998—whereas full-dose-range ERRs obtained with effect-modification estimation based on the full data (no stratification) were higher with longer follow-up: 0.502 for 1958–1987, 0.505 for 1958–1998, and 0.516 for 1958–2009. Period-stratified effect-modification–parameter estimates became more precise with longer follow-up (see Online Resource). Finally (and importantly), shorter follow-up was associated with higher correlation between the effect-modifying variables age-at-exposure and attained-age (Table 1). A more-detailed examination of this phenomenon is presented in the Online Resource.

**Table 1:** Correlation between age-at-exposure and attained-age in the LSS solid cancer mortality data

Period	1950–1985	1950–1990	1950–1995	1950–2003
Correlation	0.867	0.825	0.779	0.690

## Discussion

Estimating a minimal-significant dose level (*D*_min_) is not a strictly valid statistical procedure because the estimate is sensitive to the number and locations of the cutpoints as well as to large uncertainty that occurs with extremely narrow dose ranges coupled with small numbers of excess cases at low doses. Because of sensitivity to choice of cutpoints, the present results for mortality over 1950–2003 in Fig. 1 appear somewhat different from Fig. 1a of Walsh and Schneider (2016) despite being based on the same data. In the present analysis the stratification cutpoints from the original person-year data were used; and no further cutpoints (such as 0.03 and 0.05 Gy) were added as was done by Walsh and Schneider (2016) following Ozasa et al. (2012). Although combining strata would not pose a problem, splitting strata by choosing a non-original cutpoint not in the person-year stratification is not valid because each cell within a person-year stratum is assigned a person-year weighted mean dose that is based on individuals whose doses are above and below that mean (and hence above and below a non-original cutpoint; see the Online Resource for additional explanation and an example). Only re-stratifying the original, individual data using new cutpoints to produce a new person-year table alleviates this technical problem.

Comparing minimal-significant dose levels estimated with different follow-up periods is fraught with even greater uncertainty because of differences in background-rate–and effect-modification–parameter estimates. However, estimation of a *D*_min_ is frequently employed to assess information about low-dose risk and to infer a lowest dose at which a significant linear dose response can be established. The present definition of *D*_min_ may be conservative, but it is not sensitive to the large variation seen in the very low dose ranges. It is important to recognize that the points in Figs. 1 and 2 do not represent dose groups; they represent the slopes of linear ERR models fit to increasingly wider dose ranges that all start from zero. It is also important to bear in mind that one cannot conclude that there is no risk below the minimal-significant dose level, because lack of statistical power becomes an issue at low dose ranges. Rather, all that can be said about doses below the minimal-significant dose level is that there is insufficient statistical power to declare a significant non-zero slope if the low-dose risk is linear. In other words, lack of a significant ERR in a low-dose range may be due to a type II error—failure to reject a false null hypothesis—if the linear ERR model is true. One reviewer has pointed out that the finding of a few isolated significant dose ranges in the low-dose region might reflect nonlinearity; this is certainly possible, but due to the arbitrary nature of cutpoint selection and the potential for confounding in the low-dose range, such an interpretation is not advocated by the authors of this paper. More appropriate methods are available for assessing effects at low doses (e.g. Furukawa et al. 2016).

The slopes (ERR per Gy) in intervals associated with the *d*_*j*_’s in Figs. 1 and 2 show more-consistent patterns than those that were reported by Walsh and Schneider (2016) based on subsets of the data restricted to specific follow-up periods. This is attributed to smaller between-analysis variation in the present results, given that background estimation and effect-modification adjustment were based on a common set of data for all stratifications of ERR by follow-up period. The slope and intercept of a regression model are not independent; because the background parameters may vary according to the subset of data used, the intercept (the overall incidence or mortality) can also vary, and this would add between-period variation to the ERR estimates. This renders it difficult to compare ERR values obtained with data from different follow-up periods. More strikingly, the result of estimating effect-modification parameters with data from only a specific period illustrates how less-precise estimation of effect modification in the earlier periods had a profound impact on the ERR estimates. There was a high correlation between age at exposure *e* and attained age *a* in the early follow-up periods because, with limited follow-up time, time-since-exposure *t* = *a* − *e* has a narrow range and so the values of *e* and *a* were nearly collinear. This near-collinearity renders it difficult to accurately estimate separate age-at-exposure and attained-age effect-modification parameters in the early follow-up periods. Thus, adjustment for effect modification—and therefore the ERR estimates themselves—could be less reliable in the earlier follow-up periods. This would seem to be part of the cause of the apparent higher risks in earlier follow-up periods that Walsh and Schneider observed; there was little tendency for risk to decline with longer follow-up period when effect-modification parameters were estimated with the most-recent, complete follow-up data.

Walsh and Schneider (2016) concluded “that the main influence on *D*_min_ comes from the length of follow-up” and suggested that this is related to a decline in risk with time since exposure (owing to the fact that the ERR decreases with attained age, and attained age is correlated with time since exposure). Although RERF studies show that the ERR for solid cancer decreases with attained age, the effect is most pronounced in survivors exposed at younger ages, and excess absolute rates of solid cancer are in fact increasing with age. As reported here, the present examination of how length of follow-up contributes to changes in *D*_min_ revealed that longer follow-up provides more precise estimation of both background rates and effect-modification parameters, and the present results did not suggest that *D*_min_ is declining with longer follow-up. Therefore, the apparently higher values of *D*_min_ (and lower values of ERR at low doses) in LSS reports based on longer follow-up periods, which were discussed by Walsh and Schneider (2016), may be due to less-precise background-rate estimation and poor estimation of effect modification of the ERR in the earlier follow-up periods, rather than to accumulated time since exposure in the later periods. Information about risk modification is limited to the radiation-related excess cases, and the number of excess cases is relatively small given the skewed dose distribution. Furthermore, estimates of parameters associated with risk modifiers like attained age and age at exposure are related to estimates of background rates of cancer incidence and mortality, so avoiding bias in estimating risk-modification parameters requires precise estimation of the background effects of these factors (Cologne et al. 2002). This means that ERR estimates should be more reliable when background and effect-modification parameters are estimated with a greater amount of data.

It is cautioned, however, that the values of *D*_min_ reported here should not be construed as being definitive. The present results were based on background-rate models that are slightly different from those used in the published LSS mortality and solid-cancer incidence analyses. In particular, in the present analyses adjustment for smoking was not done with the solid-cancer incidence data, and interactions between proximal-distal–distance and city indicators were included. The latest LSS mortality and cancer incidence reports should be used as the definitive source of *D*_min_ because they are based on the most-thorough analyses, the most-complete data, and the most-precise background and effect-modification estimates. For the sex-averaged *D*_min_ with solid cancer, the latest LSS mortality report gives a value of 0.2 Gy (Ozasa et al. 2012) and the latest incidence report gives a value of 0.1 Gy (Grant et al. 2017). It should be noted, however, that these values are based on cutpoints in the person-year tabulation, not on a continuous-value estimation of minimal-significant dose range, so they should not be interpreted as being precise. The analyses of LSS mortality and cancer-incidence data are continuously evolving, so it is possible that these values might change in the future as more information becomes available and is incorporated into the analyses (such as with smoking in the latest incidence analysis).

## Conclusion

In summary, there is little evidence to suggest that results of previous LSS analyses should continue to be considered once results with longer follow-up become available. The appearance of an increase—with longer follow-up time—in the minimal-significant dose range may be due to unstable estimation of effect-modification parameters in earlier follow-up periods. Although it is true that risk has been declining with attained age, and therefore with the advance of time in the fixed cohort of A-bomb survivors, that itself is not a reason why longer follow-up should result in lower estimated risk at low doses. This is because the standard ERR models explicitly account for attained age as an effect modifier, and the reported ERR is that estimated for a survivor at attained age 70 who was exposed at age 30 regardless of length of follow-up (and as of 1985, all surviving LSS members exposed at the age of 30 had attained the age of 70). It is best to consider low-dose risks estimated from the most-recent follow-up data, which allows the most-precise adjustment for background rates and most-accurate estimates of effect modification.

## Electronic supplementary material

Below is the link to the electronic supplementary material.

Supplementary material 1 (DOCX 128 KB)
