# Supplementary material for: Effect of follow-up period on minimal-significant dose in the atomic-bomb survivor studies
Source: Radiat Environ Biophys. 2017 Nov 21;57(1):83–8. doi: 10.1007/s00411-017-0720-7 (PMC5816089; doi:10.1007/s00411-017-0720-7)
Supplement: Supplementary file 1 — Supplementary material 1 (DOCX 128 KB) [file 411_2017_720_MOESM1_ESM.docx]

**Supplementary Material**

**Effect of Follow-up Period on Minimal-Significant Dose in the Atomic-bomb Survivor Studies**

John Cologne, Dale L. Preston, Eric J. Grant, Harry M. Cullings, Kotaro Ozasa

In addition to using a common set of effect-modification parameters for the full follow-up period, analyses were performed in which the effect-modification parameters were allowed to depend on follow-up period using the same stratification as was applied to the disjoint segmented linear ERR model. The ERR estimated with effect-modification stratification was

$$\text{ERR}=\left[\left(\beta_{\text{L}1}d_{\text{L}}+\beta_{\text{U}1}d_{\text{U}}\right)\delta_{\text{P}}+\left(\beta_{\text{L}2}d_{\text{L}}+\beta_{\text{U}2}d_{\text{U}}\right)(1-\delta_{\text{P}})\right]\times\left[EM_{1}\delta_{\text{P}}+EM_{2}(1-\delta_{\text{P}})\right]$$

where $EM_{k}=e^{\tau_{k}(e-30)+\upsilon_{k}\ln(a/70)}\left(1+\sigma_{k}s\right)$, *k*∈{1,2}.

The effect-modification-parameter estimates obtained for each follow-up period stratum became progressively more precise (confidence-interval widths became progressively narrower) with longer duration of follow-up (Tables S1 and S2). With mortality (Table S1), the estimated full-dose-range ERR became progressively larger with progressively earlier end of follow-up. With incidence (Table S2), no such trend was observed, although the ERR in the earliest follow-up period (1958-1987) was noticeably higher than that in the later follow-up periods.

| **Table S1** Effect-modification parameter estimates and full-dose-range ERR by period (based on stratification) with solid cancer mortality ^a^ (95% likelihood-based CI) | | | | |
| --- | --- | --- | --- | --- |
| **Follow-up period** | **Log(age/70)**  (exponentiated) | **Age at exposure − 30**  (exponentiated) | **Sex** ^b^ | **ERR** ^c^ |
| 1950-2003 ^d^ | 0.472  (0.209, 1.12) | 0.688  (0.572, 0.808) | 0.357  (0.177, 0.530) | 0.438  (0.328, 0.550) |
| 1950-1995 | 0.615  (0.224, 1.85) | 0.678  (0.547, 0.819) | 0.320  (0.114, 0.520) | 0.474  (0.344, 0.614) |
| 1950-1990 | 0.686  (0.199, 2.70) | 0.711  (0.555, 0.891) | 0.352  (0.115, 0.585) | 0.492  (0.336, 0.675) |
| 1950-1985 | 0.943  (0.205, 4.44) | 0.683  (0.505, 0.899) | 0.322  (0.055, 0.587) | 0.544  (0.334, 0.824) |
| ^a^ The complete follow-up data (1950-2003) were used for background mortality estimation  ^b^ Sex was coded −1 for males and +1 for females to produce sex-averaged ERR estimates  ^c^ Sex-averaged ERR for a survivor with attained age 70 who was exposed at age 30  ^d^ Being the most recent follow-up period, 1950-2003 does not involve stratification of either the disjoint segmented ERR model or the effect-modification parameters | | | | |

| **Table S2** Effect-modification parameter estimates and full-dose-range ERR by period (based on stratification) with solid cancer incidence ^a^ (95% likelihood-based CI) | | | | |
| --- | --- | --- | --- | --- |
| **Follow-up period** | **Log(age/70)**  (exponentiated) | **Age at exposure − 30**  (exponentiated) | **Sex** ^b^ | **ERR** ^c^ |
| 1958-2009 ^d^ | 0.214  (0.137, 0.335) | 0.814  (0.737, 0.895) | 0.282  (0.172, 0.392) | 0.516  (0.430, 0.605) |
| 1958-1998 | 0.212  (0.123, 0.371) | 0.812  (0.720, 0.911) | 0.261  (0.133, 0.388) | 0.508  (0.416, 0.607) |
| 1958-1987 | 0.267  (0.121, 0.610) | 0.784  (0.659, 0.926) | 0.256  (0.099, 0.413) | 0.540  (0.414, 0.685) |
| ^a^ The complete follow-up data (1958-2009) were used for background incidence estimation  ^b^ Sex was coded −1 for males and +1 for females to produce sex-averaged radiation ERR estimates  ^c^ Sex-averaged ERR for a survivor with attained age 70 who was exposed at age 30  ^d^ Being the most recent follow-up period, 1958-2009 does not involve stratification of either the disjoint segmented ERR model or the effect-modification parameters | | | | |

The following plots reveal how the correlation between the two risk modifiers—attained age and age at exposure—has decreased with increasing follow-up. Because of the large number of cells in the person-year table (53782), a random sample of 2,000 cells was taken for the plots to reduce clutter. Higher correlation between these two variables in earlier follow-up periods would lead to poor estimation of effect-modification parameters due to near-collinearity. The values of Pearson correlation corresponding to the same time periods are:

1950-1955 0.998

1950-1965 0.977

1950-1975 0.934

1950-1985 0.867

1950-1995 0.779

1950-2003 0.690

As mentioned in the Discussion, it is not strictly valid to create dose ranges based on cutpoints that were not used in the original person-year table. For example, in the original LSS mortality data there are cutpoints at 0.02 and 0.04 Gy, but there is no cutpoint at 0.03 Gy. If a new cutpoint is made at 0.03 Gy based on the person-year data (not the original individual data), this will separate the person-year cells in the 0.02-0.04 Gy stratum into two sets: one having person-year mean weighted dose less than 0.03 Gy (but at least as large as 0.02 Gy) and the other having person-year mean weighted dose 0.03 Gy or larger (but less than 0.04 Gy). However, because individuals in the original 0.02-0.04 Gy stratum had weighted doses anywhere in the range 0.02-0.04 Gy, the two new strata created by the additional cutpoint at 0.03 Gy will both contain data from LSS members with doses on both sides of 0.03 Gy. Thus, the new cutpoint (0.03 Gy) cannot be considered to define a true minimally significant dose range because the range 0-0.03 will include data from individuals with doses in the range 0.03-0.04 Gy. To add a cutpoint that is not in the original person-year stratification, one would have to go back to the individual data and re-create the person-year table with the new cutpoint included in the dose stratification.
